# Supplementary material for: Ertapenem Supplemented Selective Media as a New Strategy to Distinguish β-Lactam-Resistant Enterobacterales: Application to Clinical and Wastewater Samples
Source: Antibiotics (Basel). 2023 Feb 15;12(2):392. doi: 10.3390/antibiotics12020392 (PMC9952050; doi:10.3390/antibiotics12020392)
Supplement: Supplementary file 1 [file antibiotics-12-00392-s001.zip › antibiotics-2174889-supplementary.pdf]

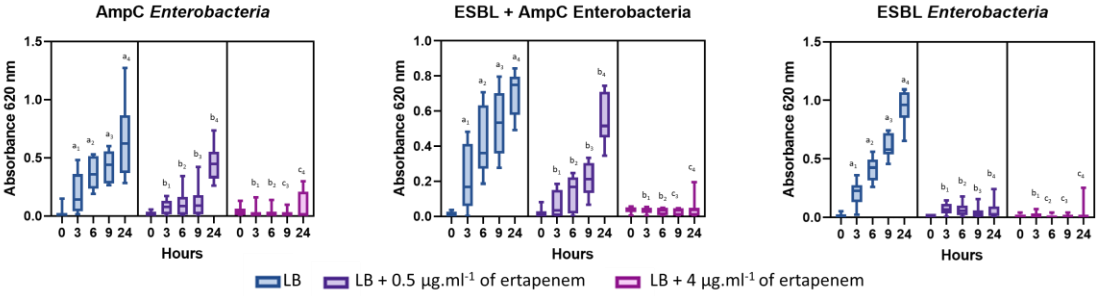


**Figure S1.** Growth overtime according to three concentrations of ertapenem for ESBL Enterobacterales (Non CRE; n=10), AmpC Enterobacterales (n=10) and ESBL + AmpC *E. cloacae* (n=13) (Non-CP-CRE). Different letters above bars indicate significant differences between ertapenem concentration as determined by Fisher's LSD test (P < 0.05). The same coefficient to a letter refers to one time point analysis.

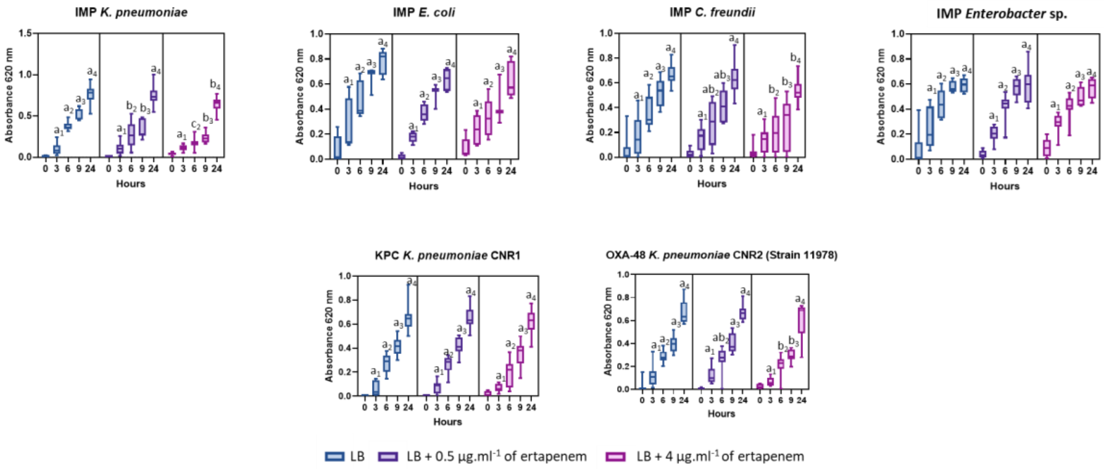


**Figure S2.** Growth over time according to ertapenem concentration (0, 0.5 or $4\mu g.ml^{-1}$) for IMP *K. pneumoniae* (n=12), IMP *E. coli* (n=5), IMP *C. freundii* (n=14), IMP *Enterobacter* sp. (n=8), KPC *K. pneumoniae* CNR1 (n=8), OXA-48 *K. pneumoniae* CNR2 (n=8). Whiskers correspond to min and max values. Different letters above bars indicate significant differences between ertapenem concentration as determined by Fisher's LSD test (P < 0.05). The same underscript number to a letter reflects time-point specific analyses.

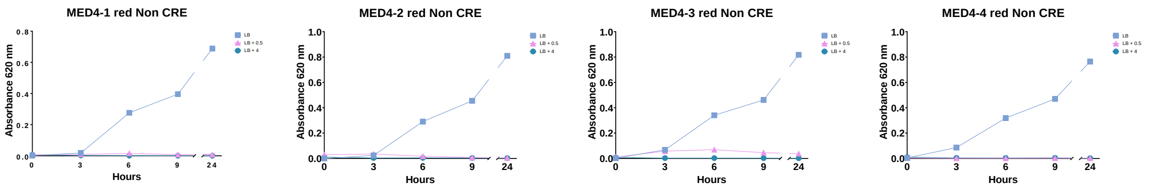

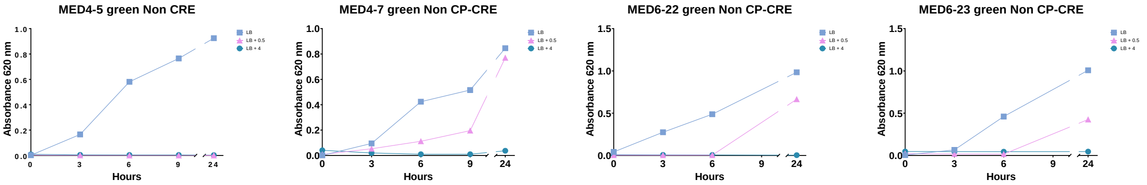


**Figure S3.** Growth overtime according to three concentrations of ertapenem for 5 colonies presumptive Non-CRE and 3 Non-CP-CRE.

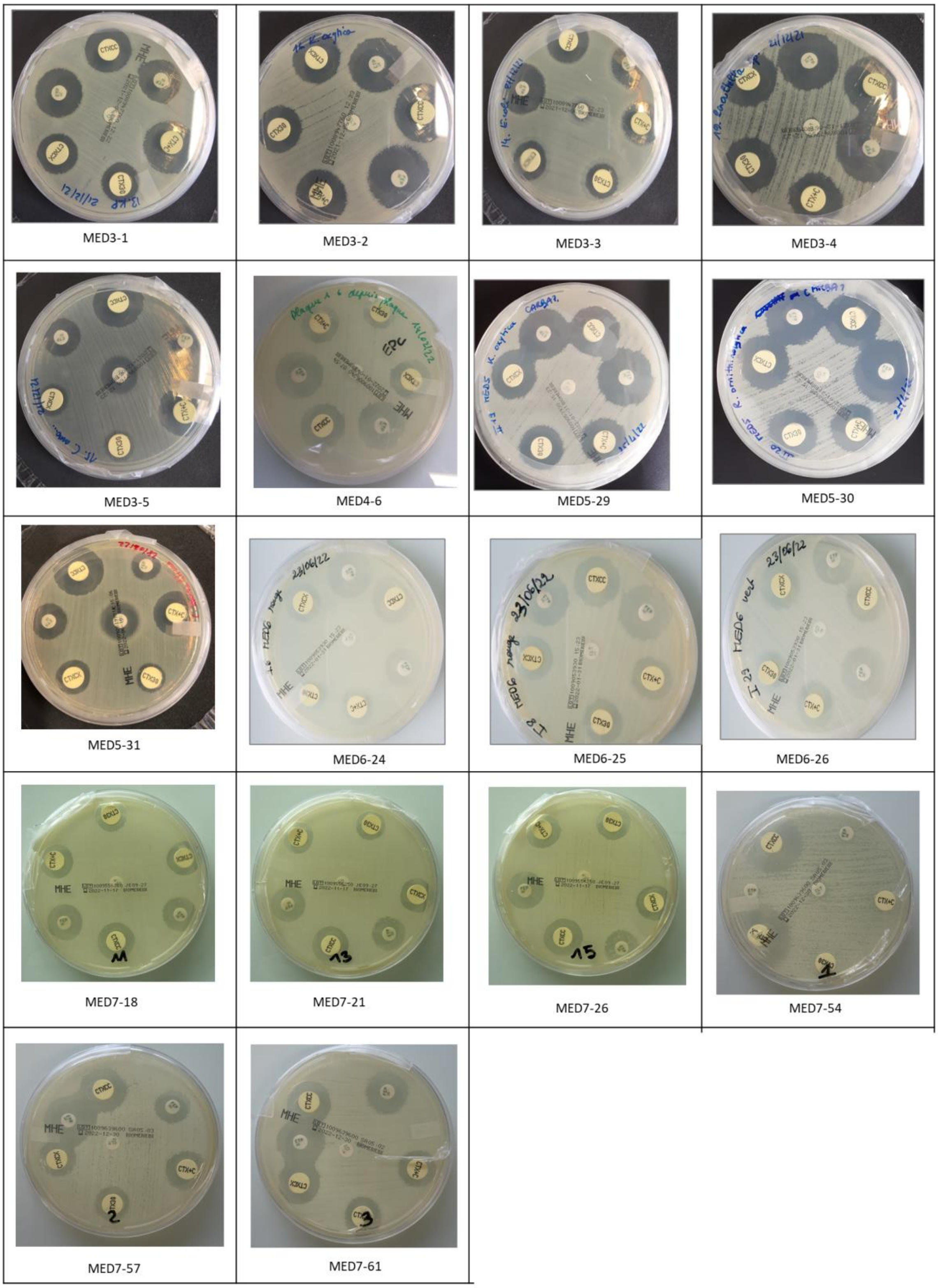


**Figure S4.** Synergy test for 18 presumptive Carbapenemase Producing Carbapenem Resistant Enterobacterales isolated from hospital effluents. CTX: cefotaxim; CTX+C: cefotaxim + clavulanic acid; CTXCX: cefotaxim + cloxacillin; CTXCC: cefotaxim + clavulanic acid + cloxacillin; FEP: cefepim; ETP: ertapenem; CLT: ceftolozan+tazobactam.
